# Supplementary material for: Prophylactic effects of probiotics or synbiotics on postoperative ileus after gastrointestinal cancer surgery: A meta-analysis of randomized controlled trials
Source: PLoS One. 2022 Mar 1;17(3):e0264759. doi: 10.1371/journal.pone.0264759 (PMC8887765; doi:10.1371/journal.pone.0264759)
Supplement: S1 Table — (DOC) [file pone.0264759.s002.doc]

**Supplementary Table 1. Electronic search strategy**

| Database | Search term (establish to April 27, 2021) | Number |
| --- | --- | --- |
| PubMed  (All fields) | #1: synbiotics OR prebiotic OR probiotics OR probiotic OR prebiotics OR synbiotic | #1: 38962 |
|  | #2: operation OR surgery | #2: 5575221 |
|  | #3: cancer OR neoplasm OR carcinoma OR tumour | #3: 4934699 |
|  | #4: #1 AND #2 AND #3 | #4: 469 |
| Embase  (All fields) | #1: synbiotics OR prebiotic OR probiotics OR probiotic OR prebiotics OR synbiotic | #1: 56395 |
|  | #2: operation OR surgery | #2: 5486220 |
|  | #3: cancer OR neoplasm OR carcinoma OR tumour | #3: 5183383 |
|  | #4: #1 AND #2 AND #3 | #4: 906 |
| Cochrane Library Trials  (All fields) | #1: synbiotics OR prebiotic OR probiotics OR probiotic OR prebiotics OR synbiotic | #1: 8555 |
|  | #2: operation OR surgery | #2: 284065 |
|  | #3: cancer OR neoplasm OR carcinoma OR tumour | #3: 225572 |
|  | #4: #1 AND #2 AND #3 | #4: 143 |
| Web of Science  (All fields) | #1: synbiotics OR prebiotic OR probiotics OR probiotic OR prebiotics OR synbiotic | #1: 50760 |
|  | #2: operation OR surgery | #2: 3978840 |
|  | #3: cancer OR neoplasm OR carcinoma OR tumour | #3: 4785108 |
|  | #4: #1 AND #2 AND #3 | #4: 472 |
|  |  |  |
