# Supplementary material for: Prophylactic effects of probiotics or synbiotics on postoperative ileus after gastrointestinal cancer surgery: A meta-analysis of randomized controlled trials
Source: PLoS One. 2022 Mar 1;17(3):e0264759. doi: 10.1371/journal.pone.0264759 (PMC8887765; doi:10.1371/journal.pone.0264759)
Supplement: S2 Table — CFU: colony forming units; C: Control group; DB: Double blind; I: Intervention group; GOS: galacto-oligosaccharides; PD: pancreatoduodenectomy; N: not available; RCT: randomized controlled trial; SC: standard care; TF: time to first flatus; TD: time to first defecation; LOP: Length of postoperative hospital stay; PI: Postoperative ileus; DS: Days to first solid diet; AB; abdominal distension; DF: days to first fluid die. (DOC) [file pone.0264759.s003.doc]

**Supplementary Table 2: Characteristics of 21 eligible studies.**

| Study | Type of study | Sample | Age | Male | Primary Disease | Type of surgery | Intervention group | Control group | Treated days (pre + post surgery) | Outcome |
| --- | --- | --- | --- | --- | --- | --- | --- | --- | --- | --- |
| Kanazawa, 2005 | RCT | I: 21  C: 23 | 64 | 29 | Biliary cancer | Combined liver and extrahepatic bile duct resection with hepaticoje junostomy | Yakult BL Seichōyaku (3 g/day) and GOS (12 g/day). Yakult BL Seichōyaku contains: 1×10^8^ living Bifidobacterium breve strain Yakult and 1×10^8^ living Lactobacillus casei strain Shirota/g | SC | Postoperative day 1–14 | LOP |
| Liu, 2010 | DB, RCT | I: 50  C: 50 | 69 | 66 | Colorectal cancer | Radical colorectomy | Encapsulated bacteria containing Lactobacillus plantarum (CGMCC No. 1258, cell count 1011 CFU⁄ g), Lactobacillus acidophilus (LA-11, cell  count 7.0 ×10^10^ CFU⁄ g) and Bifidobacterium longum (BL-88, cell count 5.0 × 10^10^ CFU⁄ g), 2 g ⁄ day | Placebo | 6 days preoperative + postoperative day 1–10 | TD, AD, DS, DF, LOP |
| Usami, 2010 | RCT | I: 32  C: 29 | 66 | 55 | Primary or metastatic liver cancer | Liver resection | Yakult BL Seichōyaku (3 g/day) and GOS (10g/day). Yakult BL Seichōyaku contains: 1×10^8^ living Bifidobacterium breve strain Yakult and 1×10^8^ living Lactobacillus casei strain Shirota/g. | SC | 14 days preoperative + postoperative day 3–14 | LOP |
| Tanaka, 2012 | RCT | I: 30  C: 34 | 62 | 51 | Esophageal cancer | Esophagectomy | Yakult BL Seichōyaku (3 g/day) and GOS (15g/day). Yakult BL Seichōyaku contains: 1×10^8^ living Bifidobacterium breve strain Yakult and 1×10^8^ living Lactobacillus casei strain Shirota/g. | SC | Postoperative day 1–21 | TD, TF |
| Zhang, 2012 | DB, RCT | I: 30  C: 30 | 65 | 24 | Colorectal cancer | Radical colorectomy | Bifid triple viable capsules, each of which contained 0.21 g (108 CFU/g) of B longum, L acidophilus and Enterococcus faecalis (one capsule 3 times a day). | Placebo | 3-5 days preoperative | PI |
| Chen, 2014 | DB, RCT | I: 35  C: 35 | N | N | Colorectal cancer | Radical colorectomy | probiotics preparation consisting of two combined live bacteria | Placebo | 5 days preoperative + postoperative day 1–7 | TD, TF, AD |
| Yokoyama, 2014 | RCT | I: 21  C: 21 | 66 | 37 | Oesophageal cancer | Oesophagectomy | One 80-ml bottle of Yakult 400, which contained at least 4 × 10^10^ living Lactobacillus casei strain Shirota; one 100-ml bottle of MILMIL-S, which contained at least 1 × 10^10^ living Bifidobacterium breve strain Yakult; and 15 g GOS (Daily) | SC | 7 days preoperative + postoperative day 1–14 | LOP |
| Komatsust, 2015 | RCT | I: 168  C: 194 | 67 | 210 | Colorectal cancer | Colorectal surgery | One 80-ml bottle of Yakult Ace, which contained at least 4 × 10^10^ living Lactobacillus casei strain Shirota with 2.5 g GOS; and one 100-ml bottle of MILMIL-S, which contained at least 1 × 10^10^ living Bifidobacterium breve strain Yakult (Daily) | SC | 7-11 days preoperative + postoperative day 2–7 | PI |
| Krebs, 2015 | DB, RCT | I: 20  C: 16 | 66 | 22 | Colorectal cancer | Colorectal surgery | One sachet consists 1011 of each of four LAB: Pediacoccus pentosaceus 5–33:3, Leuconostoc mesenteroides 32–77:1, Lactobacillus paracasei subsp paracasei 19, and Lactobacillus plantarum 2362. This makes 400 billion LAB per dose. Also included in the sachet is 2.5 g of each of the four fermentable fibres (one sachet twice a day) | Placebo | 1-3 days preoperative | TD, TF, DS |
| Liu, 2015 | DB, RCT | I: 66  C: 68 | 63 | 70 | Colorectal liver metastases | Colorectal liver metastases surgery | Encapsulated admixture of three probiotics bacteria, composed of LP (CGMCC No.1258, cell count ≥1011 CFU/g), LA-11 (cell count ≥7.0 ×10^10^ CFU/g) and BL-88 (cell count ≥5.0 × 10^10^ CFU/g) (2 g a day) | Placebo | 6 days preoperative + postoperative day 1–10 | DS, DF, AD, LOP, TD |
| Mizuta, 2015 | RCT | I: 31  C: 29 | 70 | 35 | Colorectal cancer | Colorectal cancer resection | A sachet containing 2 g of B. longum BB536 powder (approximately 5 × 10^10^ colony-forming units/2 g) daily | SC | 7-14 days preoperative + postoperative day 1–14 | LOP |
| Sommacal, 2015 | DB, RCT | I: 23  C: 23 | 60 | N | Periampullary cancer | PD | Lactobacillus acidophilus 10, 1 × 10^9^ CFU, Lactobacillus rhamnosus HS 111, 1 × 10^9^ CFU, Lactobacillus casei 10, 1 × 10^9^ CFU, Bifidobacterium bifidum, 1 × 10^9^ CFU + fructooligosaccharides 100 mg (twice daily) | Placebo | 4 days preoperative + postoperative day 1–10 | LOP |
| Tan, 2016 | DB, RCT | I: 20  C: 20 | 66 | 24 | Colorectal Cancer | Colorectal cancer surgery | An orange-flavored granular powder, containing 30 billion colony-forming units of highly compatible, acid- and bile-resistant strains of Lactobacillus acidophilus (BCMCTM12130), Lactobacillus casei (BCMCTM12313), Lactobacillus lactis (BCMCTM12451), Bifidobacterium bifidum (BCMCTM02290), Bifidobacterium longum (BCMCTM02120), and Bifidobacterium infantis (BCMCTM02129) (twice daily) | Placebo | 7 days preoperative | LOP |
| Yang, 2016 | DB, RCT | I: 30  C: 30 | 63 | 27 | Colorectal Cancer | Colorectal cancer surgery | Combined probiotics containing  Bifidobacterium longum (≥ 1.0×10^7^ CFU/g), Lactobacillus acidophilus (≥ 1.0 ×10^7^ CFU/g), and Enterococcus faecalis (≥ 1.0 ×10^7^ CFU/g) (2g, 3 times a day) | Placebo | 5 days preoperative + postoperative day 1–7 | TF, TD, LOP, DS, DF,AB |
| Zhao, 2017 | RCT | I: 40  C: 40 | 65 | 38 | Gastric cancer | Gastrectomy | Six g of live bifidobacterium and lactobacillus in tablets, 30 g of Shen Jia (daily). | SC | postoperative day 1–7 | TF, AD, LOP |
| Polakowski, 2018 | DB, RCT | I: 36  C: 37 | 60 | 39 | Colorectal cancer | Colorectal cancer surgery | Simbioflora, a dietary supplement comprising 6g of fructooligosaccharide, and the probiotics Lactobacillus acidophilus NCFM, Lactobacillus rhamnosus HN001, Lactobacillus casei LPC-37 and Bifidobacterium lactis HN019 in the concentration of 109 (6g twice daily) | Placebo | 1-7 days preoperative | LOP |
| Xie, 2018 | RCT | I: 70  C: 70 | 68 | 67 | Gastric cancer | Distal gastrectomy | Probiotics three times a day | SC | Postoperative day 1–8 | TF, LOP |
| Xu, 2018 | RCT | I: 30  C: 30 | 62 | 38 | Colorectal cancer | Colorectal cancer surgery | Bifidus-triple viable preparation daily | SC | Postoperative day 1–7 | TF |
| Bajramagic, 2019 | RCT | I: 39  C: 39 | N | N | Colorectal cancer | Colorectal cancer surgery | Probiotic capsules contains eight bacterial cultures (Lactobacillus acidophilus, Lactobacillus casei, Lactobacillus plantarum, Lactobacillus rhamnosus, Bifidobacterium lactis, Bifidobacterium bifidum, Bifidobacterium breve, Streptococcusthermophilus) (one capsule twice daily) | SC | Postoperative day 3–30 | PI |
| Park, 2020 | DB, RCT | I: 29  C: 30 | 61 | 32 | Colorectal cancer | Colorectal cancer surgery | Two g probiotic powder contained three probiotic strains (twice daily) | Placebo | 7 days preoperative + postoperative day 1–21 | PI |
| Zeng, 2020 | RCT | I: 54  C: 53 | 56 | 71 | Gastric cancer | Gastrectomy | Bifid triple viable (0.42-0.84g twice daily) | SC | Postoperative day 1–7 | TF, TD, DS |

CFU, colony forming units; C, Control group; DB, Double blind; I, Intervention group; GOS, galacto-oligosaccharides; PD, pancreatoduodenectomy N; not available; RCT, randomized controlled trial; SC, standard care; TF, time to first flatus; TD: time to first defecation; LOP: Length of postoperative hospital stay; PI: Postoperative ileus; DS: Days to first solid diet; AB; abdominal distension; DF: days to first fluid diet
